# Oral Side Effects of the Most Commonly Prescribed Drugs in Germany

**DOI:** 10.3390/dj14020083

**Published:** 2026-02-02

**Authors:** Frank Halling, Rainer Lutz, Axel Meisgeier

**Affiliations:** Department of Oral and Craniomaxillofacial Surgery, UKGM GmbH, University Hospital Marburg and Faculty of Medicine, Philipps University Marburg, 35043 Marburg, Germany; dr.halling@t-online.de (F.H.); rainer.lutz@uk-gm.de (R.L.)

**Keywords:** adverse drug reaction, database analysis, dysgeusia, Germany, oral cavity, scoring system, xerostomia

## Abstract

**Background:** The aim of this study is to investigate the potential link between the use of specific medications and oral adverse drug reactions. **Methods:** The 100 most frequently prescribed drugs in Germany in 2023 were compiled using the “PharMaAnalyst” database. According to the descriptions of adverse drug reactions (ADRs) in the patient information leaflets the ADRs were selected, analyzed and weighted with scores according to a classification system that distinguishes four groups of ADRs by frequency: ‘very common’ (4), ‘common’ (3), ‘uncommon’ (2) and ‘rare’ (1). The objective was to summarize the scores of the oral ADRs and define the ‘oral side effect score’ (OSES). **Results:** After accounting for duplication due to various brand names, 49 medications were reviewed. A total of 65% of the medications exhibited oral ADRs. The number of oral ADRs per medication ranged from one to seven. Xerostomia and dysgeusia were the most prevalent oral side effects, accounting for 37% of cases. Overall, 34% of side effects were classified as either ‘very common’ or ‘common’. The medication groups with the highest OSES were antidepressants, antibiotics and analgesics. Of the individual medications, azithromycin, gabapentin and pregabalin exhibited the highest OSES. **Conclusions:** This study provides a comprehensive overview of oral side effects associated with the 100 most frequently prescribed drugs. Patients with polypharmacy are particularly likely to experience oral side effects such as xerostomia and dysgeusia. Due to their high OSES combinations, antibiotics, analgesics or antidepressants may trigger multiple oral ADRs. It is essential that the medical community is continuously updated on pharmacological knowledge to raise awareness of oral ADRs.

## 1. Introduction

In recent decades, there has been a consistent upward trend in life expectancy in the most developed countries. From 1950 to 2021, global life expectancy at birth increased by 22.7 years, rising from 49.0 to 71.7 years [1]. However, this persistent rise in global life expectancy does not correspond with improvements in population health [2,3,4]. Consequently, the global consumption of pharmaceuticals for treating both acute and chronic diseases has continued to rise. This is especially evident among the rapidly expanding group of individuals aged 65 years and over. This demographic is predisposed to multimorbidity and the concomitant use of multiple medications (polypharmacy) [5,6]. The presence of two or more chronic health conditions is a widely adopted definition of multimorbidity [7]. In a global and regional systematic review and meta-analysis, Chowdhury et al. revealed that the overall global prevalence of multimorbidity was 37.2% [8]. The highest prevalence of multimorbidity was exhibited in South America (45.7%), followed by North America (43.1%) and Europe (39.2%). As people age, the likelihood of developing multiple persistent conditions increases. A substantial proportion of the global adult population over the age of 60 years has multimorbid conditions, with a prevalence of 51% [8]. An analysis of nearly 31 million Medicare fee-for-service beneficiaries revealed that 67% of the sample exhibited multimorbidity, a proportion which increased with age: 50% for persons under 65 years of age, 62% for those aged 65–74, and 81.5% for those aged 85 years and over [9].

The elderly population is predisposed to a higher prevalence of multiple chronic conditions, and clinical guidelines often recommend pharmacotherapy involving multiple medications [10]. The prevailing definition of polypharmacy, as outlined in the existing literature, is the administration of five or more medications daily [5]. A recently published Canadian study revealed that around 20% of older adults (aged 65–85) were living with both multimorbidity and polypharmacy [11]. The prevalence of polypharmacy is increasing worldwide. In developed countries, approximately 30% of adults aged 65 years and over take five or more medications [12,13]. A study of the medical status of elderly patients conducted in two oral and maxillofacial surgery departments in Germany found that 43% of subjects were taking multiple medications. The mean number of medications taken per patient was 4.4 [14]. Polypharmacy in combination with age-related changes in pharmacokinetics and pharmacodynamics, increases the risk of adverse drug reactions (ADR) or drug–drug interactions [15,16]. According to the European Medicines Agency (EMA) ’all noxious and unintended responses to a medicinal product’, related to any dose, should be considered adverse drug reactions. The incidence of ADRs in the elderly population is three to four times higher than in young adults. A comprehensive review of the literature reveals that the number of medications being administered is the most reliable predictor of ADR in the elderly [17]. In this context, the oral implications of prescription and non-prescription drugs are discussed and reviewed in more detail. Glick et al. have noted that all ten of the most commonly prescribed medications in the US are associated with adverse effects on the orofacial complex [18]. However, few studies have examined the quantity and frequency of oral side effects due to commonly prescribed medications [19,20,21].

Given the lack of studies that have hitherto examined the US pharmaceutical market, this study aims to address this research gap by listing, categorizing, and classifying the oral side effects associated with the 100 most frequently prescribed medications in Germany. This is particularly important because Germany was the third largest pharmaceutical market in the world in 2024, following the United States and China [22].

## 2. Materials and Methods

Healthcare professionals have a legal obligation to report adverse drug reactions to the drug commission of their respective professional organizations. German drug law stipulates that, following the approval of a medicinal product, experience regarding drug safety must be continuously and systematically collected and evaluated. PharMaAnalyst is a freely accessible analytical tool that enables the precise evaluation of all outpatient drug prescription data from around 73 million individuals insured under Germany’s statutory health insurance system (GKV). Consequently, the 3000 most frequently prescribed and highest-selling drugs of the selected year are available for individual analysis. These drugs are quality-assured by the GKV drug index at the Scientific Institute of the AOK (WIdO). When evaluating the Top 100 Preparations, all prescribed drugs are considered. In the context of pharmaceuticals, a drug is defined as an active ingredient or a combination of active ingredients from a specific pharmaceutical manufacturer. These active ingredients are grouped together for all drug variants that are marketed under the same trade name. Package size, dosage form, strength, or additional designations are not considered. According to §11 of the German Drug Law, each package leaflet must include the frequency of occurrence of every known ADR. The following classification system has been developed to organize the four most frequent groups of ADR: Very common: affecting more than 1 in 10 people (>10%), Common: affecting between 1 in 10 people to 1 in 100 people (1% to 10%), Uncommon: affecting between 1 in 100 to 1 in 1000 people (1% and 0.1%), Rare: affecting between 1 in 1000 to 1 in 10,000 people (0.1% to 0.01%). A meticulous examination of the package information was conducted to ascertain the prevalence of specific oral side effects within each category, which are arranged in alphabetical order. These side effects are angioedema, bruxism, dysgeusia, dysphagia, gingival bleeding, hypesthesia, pharyngitis, sialorrhea, sinusitis, stomatitis, thrush, tooth discoloration and xerostomia. Hypersensitivity reactions, which are defined as individual overreactions of the immune system to an antigen, are not considered.

We assigned scores to the different categories according to their clinical relevance: Four points were awarded for ‘very common’, three for ‘common’, two for ‘uncommon’ and one for ‘rare’. The sum of all scores for each drug was defined as “oral side effect score” (OSES). In the case of medication classes, we attributed all drugs to their ATC-groups according to the Anatomical Therapeutic Chemical classification (ATC), summarized all scores of the drugs belonging to this medication class and calculated the mean score for each ATC-group. The ten individual medications with the highest associated OSES were identified.

## 3. Results

**Figure 1:**
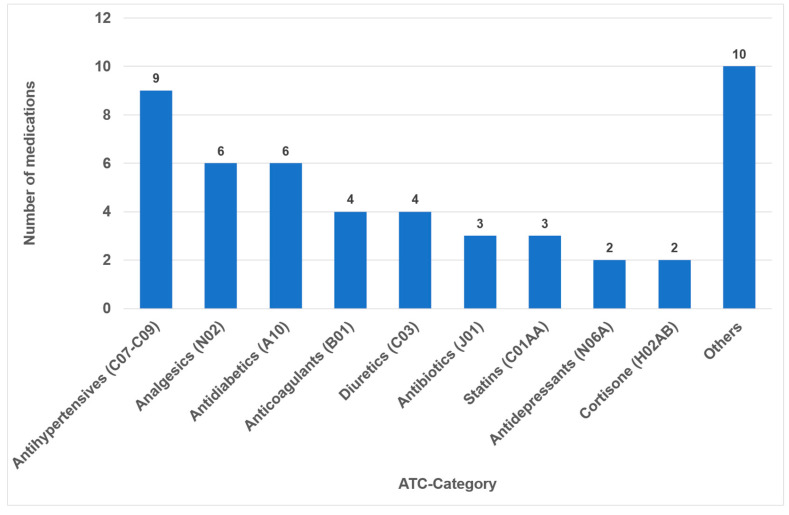
Number of drugs within the different medication classes according to ATC (*n =* 49).

In 2023, the list of the “TOP 100 Preparations” in Germany comprised 326,878,300 prescriptions and 25,249,055.1 DDD (daily defined doses), which represent a share of 44.3% percent of all prescriptions (and 52.2% percent of all DDD) within the statutory health insurance system (GKV). After accounting for duplication due to brand and generic name listings, it was found that 49 different active ingredients (medications) were covered (Supplementary Materials Table S1). Notably, medical creams and nasal drops, which collectively constitute 0.9% of the 100 most frequently prescribed medications, were not included in the study. The classes comprising the highest number of different medications were antihypertensives (nine drugs), analgesics (six drugs) and antidiabetics (six drugs) (Figure 1).

**Figure 2:**
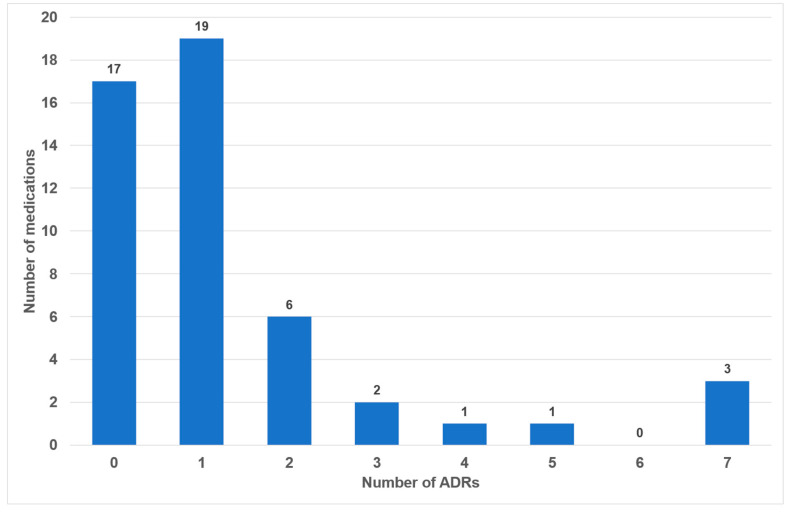
Number of medications with associated frequencies of oral side effects (*n =* 49).

**Figure 3:**
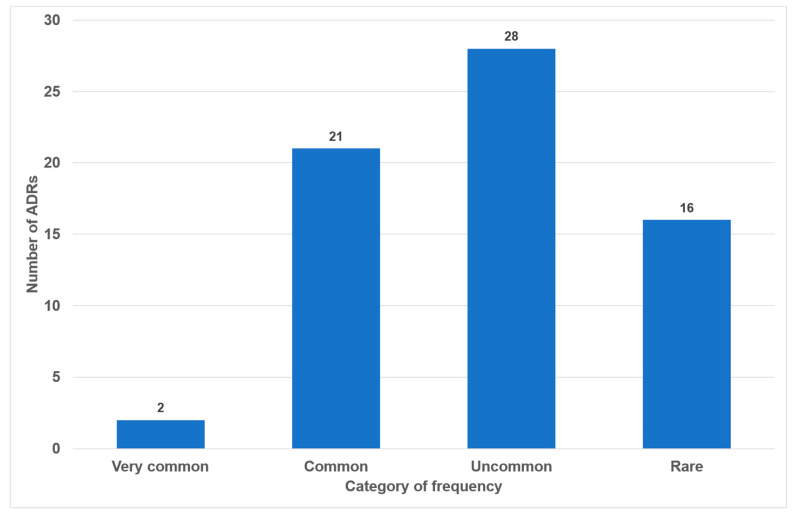
Number of oral side effects according to categories of frequency (*n =* 67).

Of the 49 drugs examined, 17 exhibited no oral side effects (35%). The remaining 25 drugs exhibited one or two adverse effects (51%). Three or more oral side effects were documented in seven of the examined drugs (14%, Figure 2). Azithromycin, gabapentin and pregabalin were found to be associated with the highest number of adverse effects (*n* = 7). Frequency distribution revealed that most oral side effects occurred in the ‘uncommon’ (*n =* 28, 42%) and ‘common’ (*n =* 21, 31%) categories. Only two oral side effects were reported as ‘very common’ (3%, Figure 3). The oral side effects were grouped into 13 different subgroups. The most frequently observed oral ADRs were xerostomia (*n =* 14), dysgeusia (*n =* 11) and angioedema (*n =* 10) (Table 1). Thus, xerostomia and dysgeusia alone account for 37.3% of all possible oral ADRs in the study group. Within the two most clinically relevant categories of side effects, this means ‘very common’ and ‘common’, xerostomia was the most prevalent (*n =* 7), followed by dysgeusia (*n =* 5) (Table 1). These two side effects alone accounted for 52% of the common and very common oral ADRs. The medication classes which are associated with the highest number of oral ADRs were analgesics, antibiotics and antihypertensives, accounting for 58.1% of cases. Only 7.5% of all oral side effects were observed in the diuretic and statin medication classes (Table 2). Furthermore, the OSES of various medication classes was calculated.

**Figure 4:**
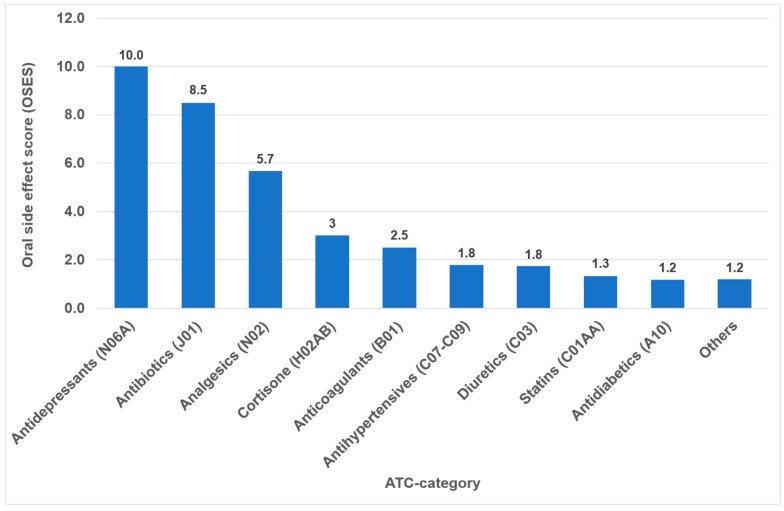
Oral side effect score (OSES) of different medication classes.

**Figure 5:**
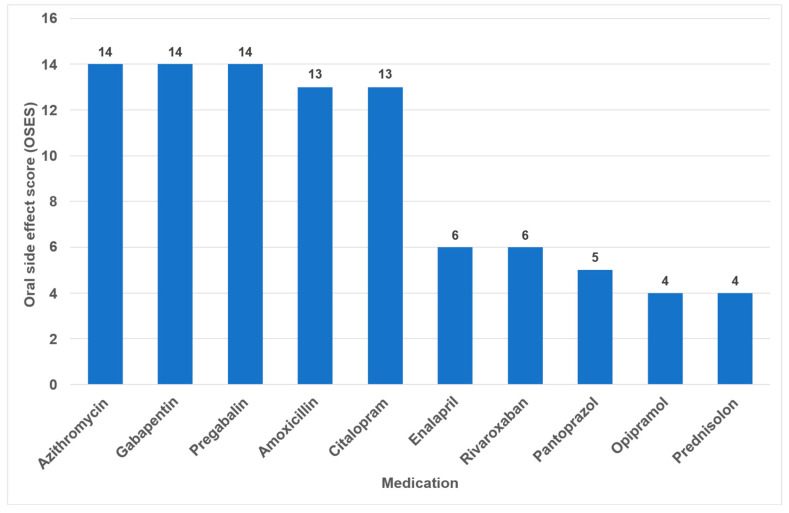
Drugs with the highest individual oral side effect scores (OSES).

Overall, the highest OSES was recorded for antidepressants (OSES = 10.0), followed by antibiotics (OSES = 8.5), analgesics (OSES = 5.7) and corticosteroids (OSES = 3.0). Antidiabetics were ranked last (OSES = 1.2, Figure 4). Regarding individual drugs with high OSESs, we found five drugs with scores above 10. These include two antibiotics (azithromycin [OSES = 14] and amoxicillin [OSES = 13]), two analgesics/anticonvulsants (gabapentin [OSES = 14] and pregabalin [OSES = 14]) and one antidepressant (citalopram [OSES = 13], Figure 5).

## 4. Discussion

All pharmaceutical agents have the potential to cause adverse effects in the human body. ADRs in dental practice have been reported to range from 5% to 10% among patients receiving dental care, indicating a considerable impact on clinical outcomes [23].

The prevalence of ADRs in elderly individuals is primarily due to the presence of multiple chronic conditions alongside polypharmacy. The elderly population is prescribed a disproportionate number of medications [17]. Projections indicate that by 2040 this demographic group will account for 40 percent of all prescriptions used [17]. The incidence of ADRs in the elderly population is reported to be three to four times higher than in young adults [17]. Patients who are prescribed five or more medications demonstrate an ADR prevalence rate of 35 percent [24]. The number of medications prescribed is the central risk factor for ADRs. Jacobsen and Chávez report an increase in ADR incidence from 6% in patients taking two medications, to 50% in those taking five, and to 100% in those taking eight or more [25].

ADRs can manifest in a wide range of clinical presentations. It has been documented that approximately 10% of adverse effects are expressed at the level of the digestive tract, affecting every part of the gastrointestinal system [26]. Drug-induced cutaneous reactions are commonly prevalent and exhibit variability in presentation. However, only a limited number of reaction patterns manifest in the oral cavity [27]. It is important to note that a side effect can only be attributed to a drug if there is close temporal proximity between its administration and the onset of the reaction. The impact of medications on oral health is well documented, with research focusing on their effects on the oral mucosa, gingiva, alveolar bone and saliva [6,18].

During our analysis of the 100 most frequently prescribed medications, 49 different medications were reviewed in total (see Figure 1 and Table S1 in the Supplementary Materials). Of these medications, 32 (65%) were associated with oral ADRs. No oral side effects were observed for 17 medications (35%) (Figure 2). This percentage is considerably higher than the 21% recorded in a comparable study of medications with no oral side effects [20]. The utilization of the classifications delineated within the German drug law enables side effects to be categorized according to their frequency of occurrence. It has been determined that 34% of all oral ADRs are classified as ‘very common’ or ‘common’, thereby indicating their clinical relevance (Figure 2). To the best of our knowledge, this is the first instance in which different oral side effects have been categorized according to their frequency of occurrence based on pharmacological data since 1994 [20]. Previous articles have historically focused on summarizing the existing literature on the subject rather than offering a comprehensive analysis of the association between registered drugs and various oral ADRs [18,28,29].

The increased life expectancy of populations has led to a heightened awareness of xerostomia as a health concern [30]. Saliva plays a vital role in maintaining oropharyngeal health, and xerostomia can significantly impact quality of life [31]. Over 500 drugs are known to cause quantitative or qualitative changes in saliva [32].

The present study the investigation revealed that 37% of all oral side effects, and 52% of those in the “common” and “uncommon” categories, are related to the medication’s impact on saliva and taste disorders (Figure 3, Table 1). This finding is consistent with the results of numerous other studies employing various methodologies, including questionnaires and interviews, dental examinations, and analyses of pharmacological databases [19,20,33,34,35]. A systematic review of the 100 most prescribed medications in the United States reported the highest prevalence of xerostomia to be more than 80 percent [21].

In the present study, 16.4% of the reviewed drugs were found to be associated with dysgeusia (Table 1). This finding is analogous to the data reported by Rademacher et al., who observed a rate of 20% [33]. Drug-induced taste disorders are significantly associated with dry mouth, with approximately 45% of the drugs known to potentially cause taste disorders also have dry mouth as an adverse drug reaction (ADR), suggesting a correlation. Possibly dysgeusia can be secondary to hyposalivation instead of being the direct effect of a drug [36]. However, the precise nature of this correlation remains challenging to ascertain, given that the term ‘dry mouth’ presumably denotes both subjective ‘xerostomia’ and objective ‘hyposalivation’ [33]. In a systematic review of drug-related dysgeusia, the authors concluded that the quality of evidence in most of the reviewed studies was low, and that a standard methodology was needed in this field [37].

In recent years, the subjects of ‘xerostomia’, ‘taste disorders’ and ‘taste loss’ have garnered increased attention in the context of quality of life and even life expectancy [38,39]. In addition to factors such as normal ageing and inadequate dietary intake, hyposalivation, xerostomia, and deficits in taste or smell perception are consequences of high medication intake and polypharmacy [38,40,41,42]. Because dysgeusia and dysosmia were reported for several drug classes with different indications, it seems reasonable that the mechanism is multifactorial and may be a combination of drug–receptor inhibition, alteration of neurotransmitter function, disturbance of action potentials in neurons, and dysfunctional sensory modulation in the brain [43]. A clear relationship has been demonstrated between the use of anticholinergic drugs or anticholinergic burden and the presence of xerostomia [44,45]. According to a comprehensive Swedish survey, the average prevalence of dry mouth was 32.1% among medicated subjects and 16.9% among non-medicated subjects. Concomitant intake of five medications resulted in a 50 percent prevalence of xerostomia [44]. Furthermore, oral candidiasis, mucosal alterations, an increased risk of caries and dysphagia are also major side effects of drugs that are associated with dry mouth [18,29,46].

The identification of the most anticholinergic medications was the object of systematic reviews and meta-analyses [47,48]. The findings indicate that especially psycholeptics and antidepressants such as amitriptyline, chlorpromazine, duloxetine and escitalopram are particularly associated with a high risk for xerostomia [47]. Due to their significant impact on saliva and taste, antidepressants exhibited the highest OSES in our study (Figure 4). Consequently, citalopram and imipramine are among the ten drugs with the highest OSES (Figure 5).

Angioedema was the third most common oral ADR in the present analysis (Table 1). Hereditary angioedema is a rare autosomal dominant genetic disorder that usually results from a decreased level of functional C1-INH. Angiotensin-converting enzyme (ACE)-induced angioedema occurs more frequently [49,50]. Medication-induced angioedema has been observed in patients treated with various medications, most commonly those that act as blockers of the renin–angiotensin–aldosterone system, like ACE inhibitors or angiotensin-II-receptor blockers [27,51]. The primary concern associated with angioedema is the potential for swelling of the tongue, the larynx and the trachea, which can result in airway obstruction and, in severe cases, death. This is the reason for the high mortality rate (30%) in patients who remain undiagnosed or are not managed properly [50]. Although this ADR was not classified as ’very common’ or ’common’ (Table 1), its clinical significance should not be underestimated, given its potential to be life-threatening. The present study identified angioedema as a potential adverse effect of ten drugs belonging to seven distinct medication groups (Table 1). According to the results of an Italian survey based on national pharmacovigilance data, the most frequently reported ADRs regarding the oropharyngeal region were characterized by throat tightness and angioedema [52]. These data highlight the important role of general dental practitioners in recognizing and managing this potentially life-threatening condition early on.

In the present analysis, antihypertensive medications were the most prevalent group of medications among the 100 most prescribed individual drugs, accounting for 18.4% (Figure 1). As in the case in Germany, antihypertensives play a significant role in pharmacotherapy in all industrialized nations [53,54]. Relative to their clinical significance, the number of their oral ADRs for this medication group is low (Table 2). In the context of OSES, antihypertensives only rank sixth among medication groups (Figure 4). Of the ten medications with the highest OSES, only one antihypertensive drug, enalapril, was identified with an OSES of 6 (Figure 5). The same observation applies to another pharmaceutical group with clinical relevance: antidiabetic medications. These drugs are the third most frequently prescribed (Figure 1), yet their OSES remains very low, resulting in their lowest ranking (Figure 4).

Analyzing the three medication groups with the highest number of oral ADRs in detail (Table 2), there are only two medications in each group with strikingly high OSES: in the case of analgesics, gabapentin and pregabalin, regarding antibiotics, azithromycin and amoxicillin, and referring to antihypertensives, enalapril and ramipril. Therefore, higher OSES seem not to be a “group-effect” typical for a special medication class but obviously depend mainly on the active ingredient of the drugs. Some impressive dental side effects like calcium channel blocker-induced gingival enlargement, are not mentioned as an ADR, because in total the incidence of these effects is very low [18]. As can be seen from Table 2 and Figure 4 and Figure 5, antibiotics and analgesics have a significant impact on the emergence of oral ADRs. Despite the limited number of antibiotics included in the analysis—namely amoxicillin, azithromycin and cefuroxime—these antibiotics were associated with the second highest OSES of all medication groups (Figure 4). Azithromycin and amoxicillin are ranked first and fourth, respectively, in our top 10 list of OSES (Figure 5). Xerostomia, dysgeusia, thrush and stomatitis were the most prevalent potential side effects that occurred in this medication group, as dry mouth may propagate oral candidiasis, which may also be caused by treatment with antibiotics, immunosuppressants or corticosteroids [55]. It is noteworthy that the prevalence of oral adverse effects resulting from antibiotic treatment is seldom documented in the existing literature. The existing literature primarily focuses on manifestations such as soft tissue involvement or thrush [56,57]. It is possible that the brief duration of administration signifies the adverse effects, reduced clinical relevance. This could be a contributor to the underreporting of ADRs. However, this assertion is not applicable to analgesics, which are frequently prescribed for extended periods and in various ways. Analgesics play the most important role in terms of the number of oral ADRs and their proportion of all oral ADRs (Table 2). Moreover, they rank second in terms of their OSES (Figure 4). This is particularly due to gabapentin and pregabalin, which are primarily used to treat neuropathic pain and also for partial seizures. In 2023, the number of prescriptions for these two drugs reached approximately 3.3 million (Supplementary Materials Table S1). Remarkably, the number of pregabalin prescriptions in Germany has dramatically increased in recent years [58,59,60]. In the context of our study, azithromycin, gabapentin and pregabalin exhibited the highest OSES alongside each other (Figure 5). This phenomenon is thought to be due to the fact that both medications have been associated with a variety of adverse effects, including xerostomia and pharyngitis. Aphthous-like ulcerations and stomatitis are more typical side effects of prostaglandin-inhibiting analgesics such as nonsteroidal anti-inflammatory drugs (NSAIDs) like ibuprofen [6]. Furthermore, they have been identified as the most prevalent causative agent in the induction of oral lichen planus [61]. A multivariate paired analysis revealed a significant association between NSAIDs combined with antihypertensive beta blockers and the development of aphthous stomatitis [62]. In the context of orofacial infections, it is crucial for dental practitioners to acknowledge the potential risk for oral ADRs associated with the combination of amoxicillin and ibuprofen, which are two of the most commonly prescribed medications in Germany [63].

As indicated by the results of an Australian study, we also found that patient information leaflets relating to oral adverse drug reactions (ADRs) were difficult to locate in Germany. This may be because there is no dedicated section for oral or dental ADRs, and the sections are applied inconsistently [64]. Furthermore, the patient information leaflets contained limited information regarding the duration, severity, reversibility, or recurrence of ADRs. To enhance the visibility of oral ADRs, an additional section for these effects should be introduced in the leaflets. At the same time, all healthcare providers should be instructed to correctly interpret the statements about the frequency of side effects in drug information leaflets. In a survey conducted in Germany, a select group of professionals, including doctors, pharmacists and lawyers, were asked to explain their interpretation of the terms ‘common’, ‘uncommon’ and ‘rare’ as they relate to medical literature. The results of this survey indicated that a significant proportion of the participants lacked a comprehensive understanding of these concepts, suggesting a need to improve the clarity and accuracy of patient education materials [65].

It is imperative to acknowledge the potential limitations of this study. Firstly, the scope of the study was delimited to the analysis of the 100 most frequently prescribed drugs in Germany in 2023. After the elimination of duplicates resulting from various brand names, the final number of medications was reduced to 49. While these data reflect the situation in only one country and in one specific year, they encompass nearly half of a significant industrialized nation’s pharmaceutical market. Secondly, the examined data do not comprise the entire prescription-only drug market; rather, they pertain exclusively to prescriptions issued to individuals insured under the statutory health insurance system. Notwithstanding, the data can be regarded as representative given that 89% of all employees in Germany are covered by the statutory health insurance [59]. Thirdly, it is well-documented that underreporting of ADRs among physicians and dental professionals is a common phenomenon [66,67]. It is noteworthy that physicians tend to report serious adverse drug reactions (ADRs) more frequently and in greater detail compared to non-serious ADRs. This phenomenon is particularly intriguing given that oral ADRs are often less severe and rarely life-threatening [68]. Fourthly, it was not possible in this study to distinguish between the effects of the medication and the effects of the underlying disease being treated in this study; the reported side effects may be attributed to either the medication or the underlying medical condition. Fifthly and lastly, because we performed an analysis of commonly prescribed drugs, there are no data or comments on rarely prescribed medication classes with significant effects on the oral cavity, such as anticancer drugs or immunosuppressives [69,70].

Oral healthcare professionals frequently encounter drugs that affect the oral region. According to the German professional code of conduct for dentists, there is an obligation to report ADRs. Unfortunately, the number of oral side effects reported to the dental drug commission has remained very low for many years, fluctuating around 100 per year. The most recent publication, which contains data from 2017, revealed that 46% of reported ADRs were related to the skin, with no distinction made between intra- and extraoral manifestations [63]. It is the responsibility of prescribers to periodically review the efficacy of medications and to remain vigilant for ADRs and their contributing risk factors [16]. The presented data demonstrate that xerostomia, dysgeusia and other oral side effects are attributable to a variety of commonly prescribed medications. When a patient presents with one or more of these complaints, it is imperative to first identify the potential triggering medication(s). Subsequently, modification or alteration of the pharmacotherapy should be made in consultation with the treating physician. Dental practitioners as well as physicians should be trained in active surveillance and reporting of oral ADRs throughout their professional lives [21]. Immediate reporting of ADRs in the oral cavity has been shown to improve care quality and to protect patients’ well-being, as patient safety is paramount.

## Figures and Tables

**Table 1:** Oral side effects in total (*n =* 67) and share in the categories ‘very common’ and ‘common’ (*n =* 23).

Oral Side Effects	Oral ADRs in All Categories		Oral ADRs in the Categories ‘Very Common’ and ‘Common’	
	*n*	Share in %	*n*	Share in %
Xerostomia	14	20.9	7	30.4
Dysgeusia	11	16.4	5	21.8
Angioedema	10	14.9	-	-
Stomatitis	7	10.4	2	8.7
Thrush	5	7.4	2	8.7
Sialorrhea	4	6.0	1	4.3
Pharyngitis	3	4.5	2	8.7
Gingival bleeding	3	4.5	2	8.7
Dysphagia	3	4.5	-	-
Tooth discoloration	2	3.0	-	-
Sinusitis	2	3.0	2	8.7
Hypesthesia	2	3.0	-	-
Bruxism	1	1.5	-	-

**Table 2:** Number of oral side effects associated with different medication classes.

Medication Classes (ATC-Category)	Medications (*n =* 49)		Oral ADRs (*n =* 67)	
	*n*	Share in %	*n*	Share in %
Analgesics (N02)	6	12.2	17	25.3
Antibiotics (J01)	3	6.1	13	19.4
Antihypertensives (C07-C09)	9	18.4	9	13.4
Others	10	20.4	7	10.4
Antidepressants (N06A)	2	4.1	6	9
Anticoagulants (B01)	4	8.2	4	6
Antidiabetics (A10)	6	12.2	3	4.5
Cortisone (H02AB)	2	4.1	3	4.5
Diuretics (C03)	4	8.2	3	4.5
Statins (C01AA)	3	6.1	2	3

## Data Availability

The original contributions presented in this study are included in the article. Further inquiries can be directed to the corresponding author.

## Supplementary Materials

The following supporting information can be downloaded at https://www.mdpi.com/article/10.3390/dj14020083/s1, Table S1: Top 100 most prescribed drugs in Germany according to PharMaAnalyst in Germany in the year 2023. After accounting for duplications resulting from brand and generic name listings, the number of different active ingredients (“medications”) covered was found to be 49.
