# Supplementary material for: Oral Side Effects of the Most Commonly Prescribed Drugs in Germany
Source: Dent J (Basel). 2026 Feb 2;14(2):83. doi: 10.3390/dj14020083 (PMC12939356; doi:10.3390/dj14020083)
Supplement: Supplementary file 1 [file dentistry-14-00083-s001.zip › dentistry-4035112-supplementary.pdf]

Supplementary Information File for:

Oral Side Effects of the Most Commonly Prescribed Drugs in Germany

Frank Halling, Rainer Lutz and Axel Meisgeier *

Department of Oral and Craniomaxillofacial Surgery, UKGM GmbH, University Hospital Marburg and Faculty of Medicine, Philipps University Marburg, 35043 Marburg, Germany; dr.halling@t-online.de (F.H.); rainer.lutz@uk-gm.de (R.L.)
* Correspondence: axel.meisgeier@med.uni-marburg.de

This document includes Supplementary table 1.

Supplementary Table S1. Top 100 most prescribed drugs in Germany according to PharMaAnalyst in Germany in the year 2023. After accounting for duplications resulting from brand and generic name listings, the number of different active ingredients ("medications") covered was found to be 49.

Ranking	Drug	Prescriptions per year	Classes
1	Dipyrone	32,275.70	Analgesic
2	Ibuprofen	24,944.20	Analgesic
3	Ramipril	23,148.20	Antihypertensive
4	L-Thyroxin	22,204.90	Other
5	Pantoprazol	19,140.60	Other
6	Bisoprolol	18,616.60	Antihypertensive
7	Candesartan	16,001.00	Antihypertensive
8	Amlodipin	14,740.60	Antihypertensive
9	Torasemid	13,112.40	Diuretic
10	Metoprolol	13,023.60	Antihypertensive
11	Atorvastatin	10,437.00	Statin
12	Metformin	8,659.40	Antidiabetic
13	Simvastatin	8,092.40	Statin
14	Salbutamol	6,490.00	Other
15	Apixaban	5,984.60	Anticoagulant
16	Tilidin	5,807.90	Analgesic
17	Lercanidipin	5,722.80	Antihypertensive
18	Amoxicillin	5,147.50	Antibiotic
19	Prednisolon	4,996.30	Cortison
20	Beclometason	3,492.40	Cortison
21	ASS	3,486.90	Anticoagulant
22	Empagliflozin	3,448.40	Antidiabetic
23	Allopurinol	3,213.20	Other
24	Azithromycin	3,193.10	Antibiotic
25	Insulin	2,835.90	Antidiabetic
26	Rivaroxaban	2,830.20	Anticoagulant
27	Dapagliflozin	2,693.70	Antidiabetic
28	Tamsulosin	2,558.00	Other
29	Xylometazolin	2,552.10	Other
30	Colecalciferol	2,489.80	Other

31	Spironolacton	2,211.50	Diuretic
32	Edoxaban	2,122.50	Anticoagulant
33	Rosuvastatin	1,954.80	Statin
34	Enalapril	1,844.20	Antihypertensive
35	Cefuroxim	1,688.40	Antibiotic
36	Hydrochlorothiazide	1,686.50	Diuretic
37	Pregabalin	1,676.80	Analgesic
38	Gabapentin	1,669.10	Analgesic
39	Noscapin	1,646.40	Other
40	Opipramol	1,640.40	Antidepressant
41	Nebivolol	1,613.10	Antihypertensive
42	Eisen	1,560.50	Other
43	Valsartan	1,543.80	Antihypertensive
44	Efeu	1,468.70	Other
45	Citalopram	1,452.80	Antidepressant
46	Diclofenac	1,433.60	Analgesic
47	Semaglutid	1,422.60	Antidiabetic
48	Chlorthalidone	1,383.90	Diuretic
49	Dulaglutid	1,292.40	Antidiabetic
**1 -49**	**Total**	**322,651.40**	
